# T18. MULTIVARIATE BRAIN ANATOMICAL DIFFERENCES IN POSITIVE AND NEGATIVE SCHIZOTYPY: PRELIMINARY RESULTS FROM THE TYPIA STUDY

**DOI:** 10.1093/schbul/sby016.294

**Published:** 2018-04-01

**Authors:** Maria F Urquijo, Eliana Faiola, Anne Ruef, Anna Kasparbauer, Inga Meyhöfer, Maria Steffens, Ulrich Ettinger, Nikolaos Koutsouleris

**Affiliations:** 1 Ludwig Maximilian University; 2 University of Bonn; 3 Ludwig Maximilians University, University Psychiatric Hospital

## Abstract

**Background:**

In schizotypy, a factor structure similar to the one observed in schizophrenia has been unraveled, being the positive and negative the most consistently replicated dimensions. Despite this fact, most of the studies on brain volume patterns in schizotypy consider it as an unitary rather than a multidimensional construct. Hence, based on previous results showing that schizophrenia and schizotypal personality traits share common neurodevelopmental patterns, it is hypothesized that brain volumetric patterns in individuals with high positive schizotypy are intrinsically different to those observed in persons reporting high negative schizotypy and to individuals with overall low schizotypal traits. The present study aims to evaluate this hypothesis using novel machine learning techniques to address the multivariate nature of psychotic diseases and the brain itself.

**Methods:**

Data from the TYPIA Study, an ongoing project conducted at the Ludwig-Maximilian University of Munich and the University of Bonn in Germany, was used to investigate whether brain volumetric patterns are distinct in healthy individuals with high positive (HPS) and high negative schizotypy (HNS) when compared to one another (HPS vs HNS) and to individuals with self-reported low schizotypy (LS vs HNS and LS vs HPS). A preliminary analysis on grey matter volumetric patterns from 29 LS (19 f., mean age: 24.6 y.), 28 HNS (20 f., mean age: 26.8 y.) and 23 HPS (17 f., mean age: 26.4 years) individuals from the general population without any current psychiatric diagnosis was performed. Group divisions were based on the introvertive anhedonia and unusual experiences subscales from the Oxford-Liverpool Inventory of Feelings and Experiences (O-LIFE). Structural images were preprocessed with a standard voxel based morphometry pipeline using the SPM-based CAT12 toolbox in Matlab. After age, sex and grey matter intracranial volume and center corrections, a linear support vector classification (SVC) algorithm was used to assess separability between the groups.

**Results:**

Our preliminary cross-validated results showed that LS and HNS can be separated with 56.0 % balanced accuracy (BAC), whereas LS vs HPS and HNS vs HPS allowed for only 42.87% and 48.8% BAC respectively. Interestingly, a post-hoc analysis comparing LS vs both high schizotypy groups merged together showed the highest BAC (59.2%). As expected, the brain differences between groups are rather small, since the sample consists fully of healthy controls. However, these results indicate that personality traits related to HNS are linked to more pronounced changes in the brain as compared to HPS. Nevertheless, schizotypy as a combination of the positive and negative dimensions allowed for a higher classification accuracy when compared to LS, supporting the notion of schizotypy as a unitary construct as observed from the post-hoc analysis. Furthermore, HNS and HPS were not separable by the algorithm, most likely due to the intrinsic heterogeneity of the construct.

**Discussion:**

Our results align with previous studies claiming that negative symptoms are associated with structural changes in the CNS whereas positive symptoms relate to changes in functioning and activation of the brain. A larger sample as well as using other data modalities will confirm the stability of our findings. Research on volumetric patterns of the brain areas related to negative symptoms in non-clinical samples might lead to a better understanding of the underlying causes of schizophrenia. Above all, our results show that investigating non-clinical expression of psychosis-like symptoms is a promising strategy to understand the prodromal stadium of schizophrenia.

